# Impact of baicalin and components of Scutellaria baicalensis on renal fibrosis of diabetic kidney disease

**DOI:** 10.3389/fphar.2024.1480626

**Published:** 2024-12-06

**Authors:** Jiarui Li, Yuan Zhuang, Guoyong Fan, Simeng Wang, Enhui Yan, Jianpeng Guo, Chi Zhang, Shicui Jiang

**Affiliations:** ^1^ Wenzhou Key Laboratory for the Diagnosis and Prevention of Diabetic Complications, The Third Affiliated Hospital of Wenzhou Medical University (Ruian People’s Hospital), Ruian, China; ^2^ College of Pharmacy, Yanbian University, Yanji, Jilin, China; ^3^ Liji Medical Research Academy, The Third Affiliated Hospital of Wenzhou Medical University (Ruian People’s Hospital), Ruian, China

**Keywords:** flavonoids, traditional Chinese medicine, diabetic nephropathy, baicalin, renal fibrosis, insulin sensitivity

## Abstract

**Background:**

Fibrosis is key in the development and progression of diabetic kidney disease (DKD). Baicalin (BA), wogonin (WGN), and wogonoside (WGS) have renoprotective effects. The mechanism of alleviation of DKD progression, by improving renal fibrosis, is unclear. This study aimed to investigate the mechanisms and effects of a *Scutellaria baicalensis* Georgi. (Lamiaceae) mixture (MIX, WGN:BA:WGS = 4:2:1) on DKD in a spontaneous DKD model.

**Methods:**

Male *db/m* mice were controls, and *db/db* mice were diabetes models. Both groups received daily oral gavage of normal saline. Treatment groups received daily oral gavage of BA or MIX (20 mg/kg) for 10 weeks. Biochemical indicators and kidney lesions were assessed. Fibrosis-related proteins were detected by immunoblotting, immunohistochemistry, and real-time fluorescence quantitative PCR.

**Results:**

MIX significantly reduced body weight (40.97 ± 1.43 vs. 42.26 ± 1.60), improved insulin sensitivity (63.70 ± 8.98 vs. 109.48 ± 0.69), lowered the renal hypertrophy index (19.81 ± 2.86 vs. 28.94 ± 0.256), and decreased blood urea nitrogen levels (7.57 ± 0.79 vs. 9.57 ± 0.38) and the urine protein/creatinine ratio (0.50 ± 0.06 vs. 0.80 ± 0.18). MIX also enhanced lipid profiles and renal function by improving renal tubular dilation, restoring renal structures, and reducing glomerulosclerosis, basal membrane thickening, and glycogen deposition. These effects were achieved by reducing the protein and gene expression of collagen II (Col-II), connective tissue growth factor, and collagen I (Col-I).

**Conclusion:**

MIX inhibits the transforming growth factor-β/Smads signaling pathway, thus alleviating renal fibrosis, and can be used to develop a treatment for DKD.

## Graphical Abstract

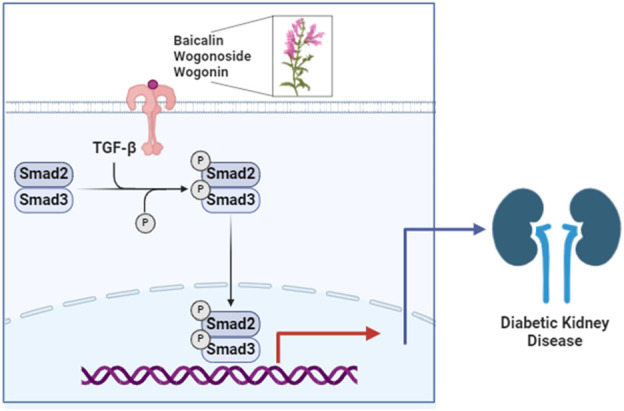

## Highlights

• MIX outperformed BA in reducing renal fibrosis markers.
• MIX significantly lowered the renal hypertrophy index in DKD mice.
• BA and MIX improved insulin sensitivity and reduced blood urea nitrogen.
• MIX inhibited TGF-β/Smad signaling, alleviating renal fibrosis.
• MIX shows potential as a natural treatment for diabetic nephropathy.

## Introduction

Diabetic kidney disease (DKD) is the main cause of end-stage lesions in diabetic patients (Aschner et al., 2021; Du et al., 2017). It is one of the most serious complications, affecting 10%–40% of patients (Adeshara et al., 2016). In China, the incidence of DKD is expected to increase in the coming decades as the prevalence of diabetes rises (Guo et al., 2016; Tang et al., 2017).

The pathogenesis of DKD involves many factors including metabolic disorders, immune-inflammatory mechanisms, oxidative stress, and genetic factors (Li et al., 2017; Lavoz et al., 2020; Meshkani and Vakili, 2016). Renal fibrosis is considered a complex and irreversible process occurring in the DKD lesions at later stages, further exacerbating disease progression. Renal fibrosis is a multifactorial dynamic process, with studies indicating the involvement of numerous factors in DKD fibrosis development including transforming growth factor-ß (TGF-ß), interleukin (IL)-1, IL-6, tumor necrosis factor-α, and oxidative stress. Research has found that NAD(P)H quinone oxidoreductase 1 mitigates diabetes-induced renal inflammation and fibrosis by modulating the Toll-like receptor 4 (TLR4)/NF-κB and TGF-ß/Smads signaling pathways (Qiu et al., 2023). Under the influence of multiple factors leading to inflammation and damage, excessive extracellular matrix deposition and the epithelial-mesenchymal transition occur, resulting in a loss of differentiated epithelial cells and their capillaries, and the accumulation of myofibroblasts and inflammatory cells, ultimately leading to structural and functional damage of the kidney (Hu et al., 2021; Yamashita and Kramann, 2024).

*Scutellaria baicalensis* Georgi. (Lamiaceae), a plant with a medicinal history spanning at least 2000 years, was first recorded around 200 AD in *Shennong’s Classic of Materia Medica* (Liao et al., 2021; Zhao et al., 2019). The roots of *S. baicalensis* are known to promote wound healing, remove heat and dampness, clear fires, detoxify and reconcile toxins, and regulate immunity. It is used for the prevention and treatment of diabetes, have the effects of improving the renal function, insulin resistance and retinopathy of type 2 diabetic patients (Lai and Li, 2024). And exhibits various pharmacological properties, including antitumor, free-radical-scavenging, antioxidant, anti-inflammatory, and antiviral activity (Shang et al., 2023). Baicalin (BA), wogonin (WGN), and wogonoside (WGS) are bioactive flavonoids found in *S. baicalensis* (Wu et al., 2014). BA has numerous health benefits, including anti-inflammatory, antibacterial, antitumor, and antioxidant activity (Liao et al., 2021; Shang et al., 2023). BA has been proposed as a potential drug for the treatment of DKD (Hu et al., 2024), alleviate podocyte damage (Ou et al., 2021), diminish renal fibrosis [ (Zhang et al., 2020; Wang H. et al., 2022)], enhance renal functionality (Yang et al., 2019), oxidative stress and inflammation (Ma et al., 2021) and provide relief in DKD scenarios. WGN mitigates glomerulopathy and podocyte injury by regulating Bcl-2-mediated crosstalk between autophagy and apoptosis (Liu et al., 2022). WGS and WGN can reduce the expression levels of TLR4 mRNA and NF-κBp65 mRNA in renal tissue. Consequently, the proteins TLR4 and NF-κBp65 are decreased, inhibiting the TLR/NF-κB signaling pathway and playing a protective role in renal tissue (Wang YR. et al., 2022).

*Scutellaria baicalensis* Georgi. (Lamiaceae) mixture (MIX, WGN:BA:WGS = 4:2:1), that using multi-chamber electrophoresis screening technology, three components that bind to the target protein were selected from a multi-component mixture of *S. baicalensis*. These components, qualitatively identified as WGN, BA, and WGS through LC-MS/MS in the multiple reaction monitoring mode, were subsequently quantified. The molar ratio of the components was 4:2:1 (v/v). Using a high glucose-induced human renal tubular epithelial cell (HK-2) model, we demonstrated that these components suppress inflammatory responses and mitigate apoptosis in high glucose-induced HK-2 cells (Suo, 2022). There is insufficient *in vivo* evidence validating the therapeutic efficacy and safety of MIX in animal models of DKD and inadequate understanding of the molecular pathways modulated by these combined components, specifically through the TGF-β/Smads signaling pathway.

In this study, we investigated the impact of multiple components of *S. baicalensis* on the TGF-β/Smads signaling pathway in the renal tissues of DKD *db/db* mice. We aimed to explore the mechanisms by which BA and MIX ameliorate DKD and provide an experimental foundation for the potential clinical applications of BA and MIX.

## Methods

### Ethics statement

All animal experiments were conducted in accordance with internationally recognized animal welfare guidelines and were approved by the Medical Ethics Committee of Experimental Animal Ethics at Ruian People’s Hospital (SYSQ-2023-008, 30 April 2023).

### Drugs

BA (>98.0% purity, catalog no. MB6698), WGN (>98.0% purity; catalog no. MB6662), and WGS (>98.0% purity; catalog no. MB6663) were purchased from Meilunbio (Dalian, China). A MIX of *S. baicalensis* was prepared using WGN, BA, and WGS in a molar ratio of 4:2:1. Isoflurane was obtained from RWD Life Science Co. Ltd. (Shenzhen, China).

### Experimental animals and treatment

*db/db* mice (males, 7 weeks, weighing 36 ± 2 g, n = 18), and *db/m* mice (males, 7 weeks, weighing 22 ± 2 g, n = 6) which were non-diabetic mice born in the same litter as the *db/db* mice, were obtained from Jiangsu Changzhou Kavins Laboratory Animal Co., Ltd. (license number: SCXK (Su) 2021-0013). They were housed in separate individually ventilated cages (temperature: 23°C ± 3°C, humidity 40%–70%, light/dark cycle 12 h). The mice had *ad libitum* access to a standard diet and water.

The *db/db* mice were divided randomly into three groups based on fasting blood glucose levels and body weight (n = 6): the *db/db* group (model of diabetes), the *db/db* mice treated with MIX group, and the BA group. The *db/m* mice (n = 6) served as the normal control group of non-diabetic mice (Jin et al., 2023). Mice in the *db/m* and *db/db* groups received normal saline, while those in the BA group received a daily dose of 20 mg/kg BA, and those in the MIX group received 20 mg/kg MIX. The three components were calculated according to a WGN: BA: WGS ratio of 4:2:1. All treatments were administered by oral gavage once daily at 10:00 a.m. for 10 weeks.

### Metabolic and biochemical parameters

The fasting blood glucose levels of the mice were assessed using a blood glucose meter and test strips once a week throughout the treatment period, with blood samples obtained from the tail tip. At 18 weeks old, the mice were weighed and placed in metabolic cages for 24 h for urine collection. The mice were anesthetized using an anesthesia machine with 1.5%–2% isoflurane. After anesthesia, blood samples were collected from the orbital venous plexus, and both kidneys were carefully removed, washed with phosphate-buffered saline, and weighed. The following parameters were measured using an automated biochemical analyzer (IDEXX Laboratories, Shanghai, China): urine protein, urine creatinine, blood urea nitrogen (BUN), cholesterol, and triglycerides.

### Intraperitoneal glucose tolerance

The mice were fasted for 12 h. An intraperitoneal injection of a glucose solution (2 g/kg, 50%) was administered. Blood samples were collected from the tail vein 0, 30, 60, and 120 min after the injection, and the corresponding blood glucose values were recorded. The area under the curve (AUC) for the glucose tolerance test was calculated (Huang et al., 2024).

### Kidney histology

The kidney tissues were fixed in 4% paraformaldehyde buffer (P1110, Beijing Solaibao Technology Co., Ltd.), embedded in paraffin, and sectioned. These sections were further processed for histological staining using hematoxylin and eosin (HE) (G1120, Beijing Solaibao Technology Co., Ltd.), Masson’s modified trichrome (G1340, Beijing Solaibao Technology Co., Ltd.), or periodic acid-Schiff (PAS) (C0142S, Beijing Solaibao Technology Co., Ltd.) and viewed under a 400× power lens.

### Immunohistochemistry staining

Following the standard deparaffinization and hydration protocol. Antigen repair was performed by microwaving 10 mM sodium citrate (pH 6.0) buffer solution for 10 min. To block endogenous peroxidase activity, the slides were incubated with 3% hydrogen peroxide (H_2_O_2_) at room temperature for 30 min. The slides were blocked with 20% normal goat serum for 1 h. The sections were incubated overnight with the appropriate primary antibody TGF-β (25 kDa, 1:500, #ab92486, Abcam, Cambridge, United Kingdom) and Smad2/3 (60 kDa, 1:500, #3700, CST, Danvers, MA, United States) were applied to the slides and left overnight at 4°C. (Negative control: no primary antibody was added during the staining process). DAB color development (DAB chromogenic reagent for histochemical kit; G1212; Wuhan servicebio technology CO.,LTD.) and counterstained with hematoxylin (G1004; G1039; G1040; Wuhan servicebio technology CO.,LTD.). Images were captured at a magnification of ×400 and analyzed Immunohistochemistry positive area using ImagePro Plus 6.0 quantitative software (NIH, Bethesda, MD, United States).

### RT-qPCR

Total RNA was extracted from mouse kidney tissues using the RNAiso Plus reagent following the standard protocol (MF846, Mei5 Biotechnology, Beijing, China). The cDNA was reverse-transcribed (MF012, Mei5 Biotechnology, Beijing, China). RT-qPCR (MF049, Mei5 Biotechnology, Beijing, China) was performed on the QuantStudio™ 3 system (Thermo Fisher Scientific, Waltham, MA, United States). The mRNA levels of the target genes were normalized and analyzed using the 2^−ΔΔCT^ method. The primer sequences are listed in Table 1.

**TABLE 1:** Primer sequences.

Gene	Primer sequence
Collagen Ⅰ (Forward)	CTG​ACG​CAT​GGC​CAA​GAA​GA
Collagen Ⅰ (Reverse)	TAC​CTC​GGG​TTT​CCA​CGT​CT
β-Actin (Forward)	ATC​ACT​ATT​GGC​AAC​GAG​CGG​TTC
β-Actin (Reverse)	CAG​CAC​TGT​GTT​GGC​ATA​GAG​GTC

### Western blot assays

Renal tissues (approximately 100 mg) were collected and lysed in radioimmunoprecipitation assay buffer (P0013B, Beyotime Biotechnology Co., Ltd.). The lysates were centrifuged at 4°C and 12,000 g for 15 min, and the resulting supernatants were collected. Total protein was extracted from the supernatant, and the protein concentration was ascertained using a bicinchoninic acid assay (BCA, P0010 Beyotime Biotechnology Co., Ltd.). Equivalent amounts of protein (35 μg/lane) were separated by sodium dodecyl sulfate-polyacrylamide gel electrophoresis (SDS-PAGE, P0010, Beyotime Biotechnology Co., Ltd.) and transferred to polyvinylidene difluoride membranes by electroblotting.

The membranes were blocked in 5% (w/v) nonfat milk at room temperature for 1 h. They were then incubated overnight at 4°C with specific primary antibodies against TGF-β (25kDa, 1:1,000 dilution; #ab92486, Abcam, Cambridge, United Kingdom), Smad2/3 (60 kDa, 1:1,000 dilution; #12470, CST, Danvers, MA, United States), collagen I (Col-Ⅰ) (220 kDa1:1,000 dilution, #72026 CST, Danvers, MA, United States), collagen II (Col-Ⅱ) (200 kDa, 1:1,000 dilution, #43306S, CST, Danvers, MA, United States), and connective tissue growth factor (CTGF) (35 kDa, 1:1,000 dilution, #86641S, CST, Danvers, MA, United States). The internal control β-actin/GAPDH calibration was used to correct the errors in the quantification and loading of protein samples to ensure the accuracy of the experimental results. After washing with tris-buffered saline/0.1% Tween 20 (TBST), the membranes were incubated with the appropriate secondary antibody at room temperature for 1 h. Following washing with TBST, the membranes were stained with an enhanced chemiluminescence reagent and visualized using a gel imaging system. ImagePro Plus 6.0 was used to analyze the gray scale of the obtained strips.

### Statistical analyses

Data analysis was conducted using SPSS version 22.0. Results are presented as mean ± standard deviation. Differences among multiple sample groups were assessed using one-way analysis of variance. Pairwise comparisons between groups with homogeneous variances were performed using the Bonferroni method, while Tamhane’s T2 test was employed for groups with heterogeneous variances. *p* < 0.05 was considered statistically significant.

## Results

### Effect of baicalin and MIX on weight, blood sugar, and glucose tolerance of db/db mice

During the experiment, both the *db/m* and *db/db* groups exhibited significant increases in average body weight. However, after 4 weeks of treatment, the *db/db* MIX treatment group showed a marked decrease in body weight, surpassing the decrease observed in the *db/db* BA group. The *db/db* group demonstrated a notable elevation in blood glucose levels compared to the *db/m* group. Neither BA nor MIX administration in the *db/db* group reduced the blood glucose level. This indicates that both BA and MIX were ineffective in mitigating the onset and progression of DKD by lowering blood glucose levels.

**FIGURE 1:**
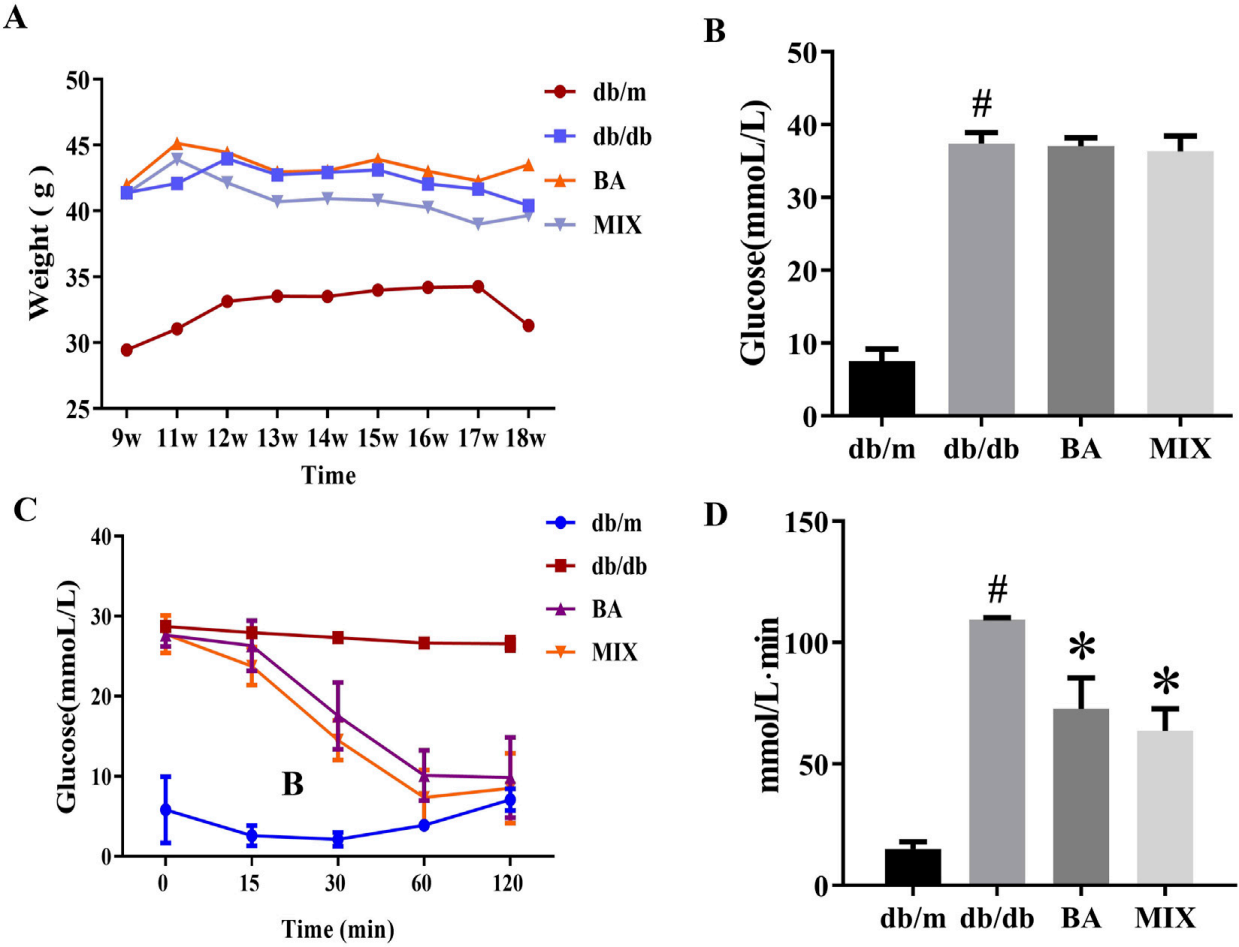
Changes in body weight, blood glucose, and glucose tolerance in each group **(A)** The weight change (g) of each group over the study period. **(B)** The blood glucose levels (mmol/L) measured at the end of the study. **(C)** The time trend of blood glucose levels (mmol/L) after insulin injection. **(D)** The area under the curve of the insulin tolerance test for each group (n = 6, compared to the *db/m* group, #*p* < 0.05; compared to the *db/db* group, *p < 0.05). Abbreviations: db/m: normal control group of non-diabetic mice, db/db: model of diabetes, BA: baicalin, MIX: multi-component mixture.

In the *db/m* group, blood glucose levels gradually decreased after intraperitoneal insulin injections and reached their lowest value 30 min after injection. In contrast, blood glucose levels in the *db/db* group did not change significantly after insulin injection, indicating a marked decrease in insulin sensitivity and the presence of insulin resistance. The AUC of the insulin tolerance test also increased significantly in the *db/db* group compared to the control group. In the BA and MIX treatment groups, blood glucose levels decreased after intraperitoneal insulin injection, reaching their lowest values at 60 min. This indicates that BA and MIX restored insulin sensitivity in mice. Among the treatment groups, the MIX group demonstrated the greatest improvement in glucose and insulin tolerance, as indicated by the insulin tolerance test AUC values shown in Figure 1.

### Effect of baicalin and MIX on renal function and serum biochemical indicators in db/db mice

**FIGURE 2:**
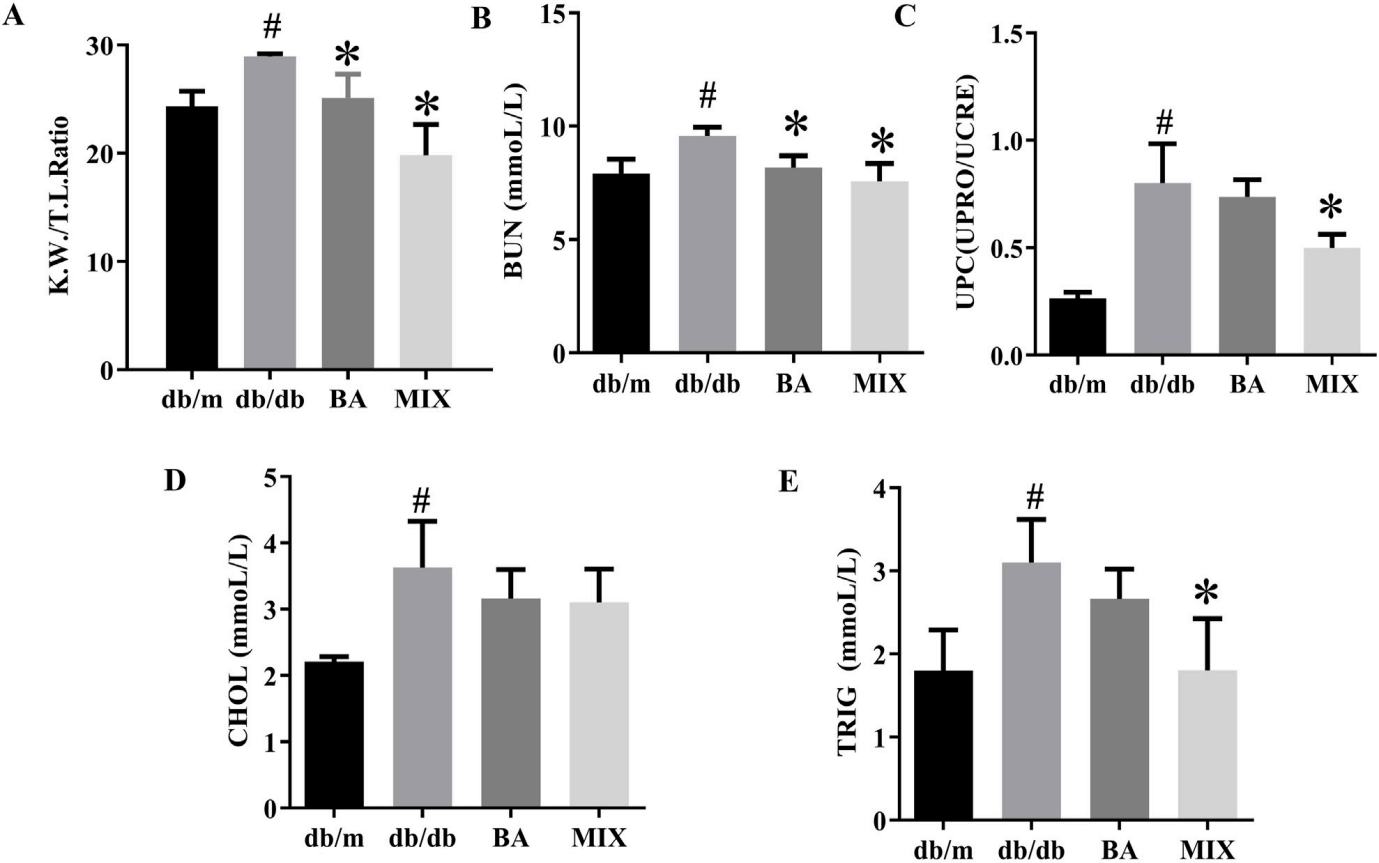
Effect on renal function, and serum biochemical indicators, in each group of type 2 diabetic mice **(A)** The ratio of renal weight to tibial length. **(B)** Urea nitrogen. **(C)** Ratio of urinary protein to urinary creatinine. **(D)** Cholesterol level. **(E)** Triglyceride level (n = 6, compared to the db/m group, #*p* < 0.05; compared to the db/db group, *p < 0.05). Abbreviations: K.W: renal weight, T. I: tibial length, BUN: blood urea nitrogen, UPRO: urinary protein, UCRE: urinary creatinine, CHOL: cholesterol, TRIG: triglyceride, db/m: normal control group of non-diabetic mice, db/db: model of diabetes, BA: baicalin, MIX: multi-component mixture, UPC: ratio of urinary protein to urinary creatinine.

The ratio of kidney weight to tibial length in mice serves as an indicator of renal hypertrophy. Compared to the *db/m* group, the *db/db* group exhibited a significant increase in the renal hypertrophy index. However, the BA and MIX treatment groups significantly reduced the renal hypertrophy index compared with the *db/db* group. In particular, the MIX treatment group showed a more substantial decrease in the renal hypertrophy index than the BA treatment group did, as shown in Figure 2A.

The *db/db* group showed a substantial increase in BUN levels compared with the *db/m* group. In contrast, the BA and MIX treatment groups exhibited varying degrees of reduction in BUN levels compared with the *db/db* group, suggesting improved renal function. Among the treatment groups, the MIX treatment group showed a significant decrease in BUN levels compared with the BA treatment group (Figure 2B).

The urinary protein-to-creatinine ratio was significantly higher in the *db/db* group than in the *db/m* group. However, the treatment groups demonstrated varying degrees of reduction in this ratio, indicative of improved renal function. The MIX treatment group exhibited the most pronounced reduction in the urinary protein-to-creatinine ratio, especially when compared to the BA treatment group (Figure 2C). These findings suggest that MIX enhanced renal filtration and alleviated the progression of DKD.

The *db/db* group demonstrated a significant increase in cholesterol (CHOL) and triglyceride (TRIG) levels compared with the control group. However, the treatment groups showed varying degrees of reduction in TRIG and CHOL levels compared with the *db/db* group, indicating a beneficial effect. In particular, the MIX treatment group exhibited a significant decrease in TRIG levels compared with the *db/db* group. The results are shown in Figure 2E.

### Effect of baicalin and MIX on renal pathological morphology of db/db mice

HE and PAS staining revealed a well-preserved renal tissue structure in the *db/m* group, with normal glomerular size and morphology and healthy interstitial space without mesangial proliferation. The nuclear membrane boundaries were clear and showed no abnormalities and the renal tubules maintained their integrity and compact arrangement, with no anomalies detected in the interstitium. In contrast, the *db/db* group exhibited substantial abnormalities, including extensive strip-shaped blank areas, significant reduction in nuclei, loss of renal tubular shape, noticeable tubular dilation, epithelial cell apoptosis and necrosis, cell loss, and abnormal changes in the renal interstitium. Following the administration of various doses of BA and MIX, pathological improvements were observed to varying degrees, with a reduction in interstitial fibrosis. The BA treatment group showed more pronounced improvement than the MIX treatment group did, indicating greater efficacy in mitigating these pathological changes.

**FIGURE 3:**
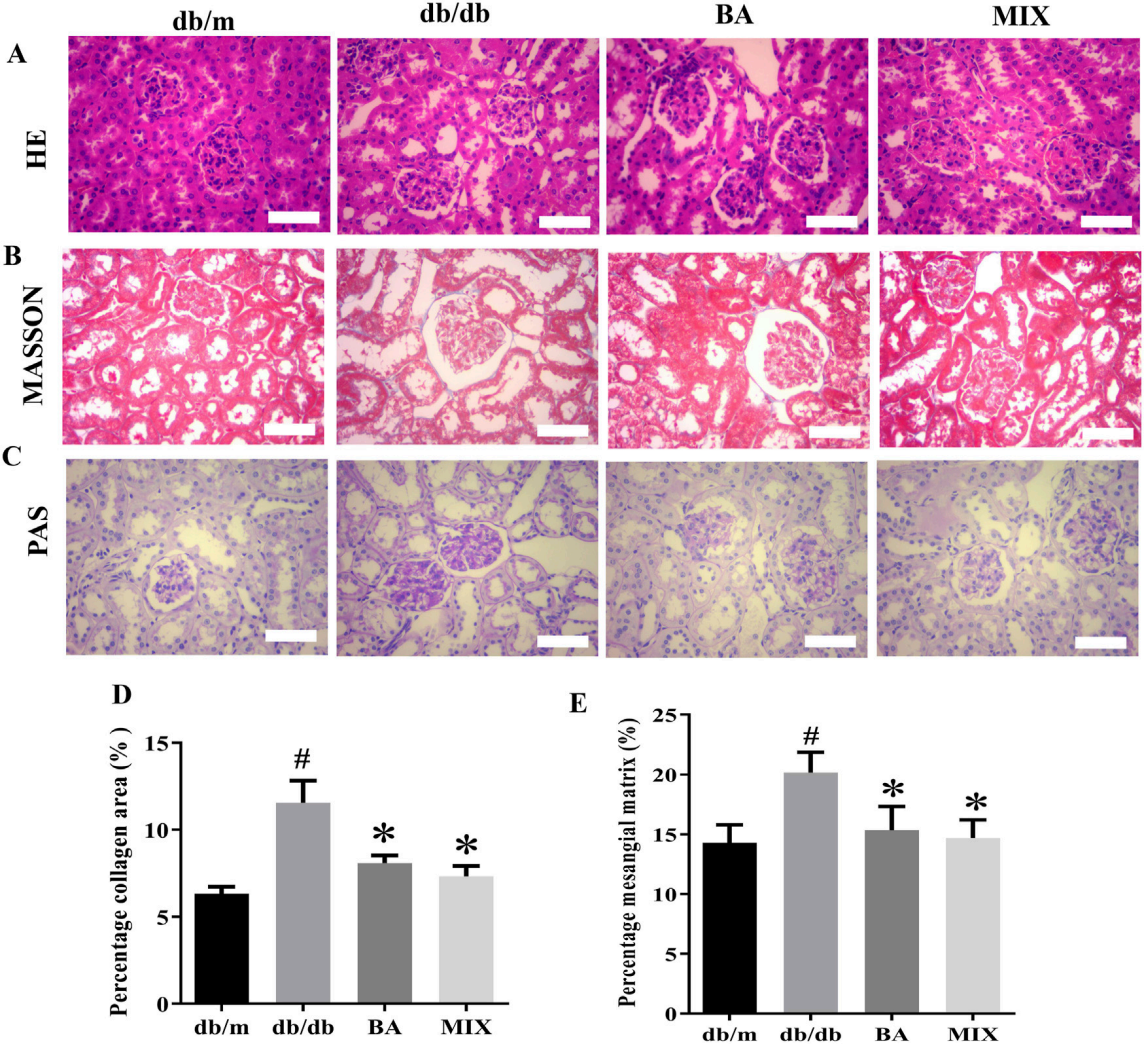
Photomicrographs of kidney sections from each group of mice, stained with hematoxylin and eosin **(A)** Masson’s trichome **(B)** and periodic acid-Schiff **(C)** observed under a light microscope (×400). **(D)** Changes in the glomerular area are seen with Masson’s trichome staining. **(E)** The percentage of the mesangial matrix observed with PAS staining (n = 6, compared to the *db/m* group, #*p* < 0.05; compared to the *db/db* group, *p < 0.05). Abbreviations: HE: hematoxylin and eosin, PAS: periodic acid-Schiff, db/m: normal control group of non-diabetic mice, db/db: model of diabetes, BA: baicalin, MIX: multi-component mixture.

In the *db/m* group, the boundaries of the kidney cells remained distinct. The glomeruli showed no significant signs of sclerosis, with no collagen production or minimal filamentous deposition. In contrast, in the *db/db* group, the glomeruli exhibited marked fibrosis, intercellular congestion, substantial accumulation of collagen fibers around the renal tubules, widening of the renal interstitium, a significant increase in collagen fibers, the presence of protein casts, and diffuse distribution throughout the renal interstitium. The areas of collagen deposition and fibrosis were significantly reduced in each treatment group compared to the model group. Only a small number of filamentous fibers were observed in the BA and MIX treatment groups, with the most substantial improvement seen in these groups, as shown in Figure 3.

Compared to the *db/m* group, the glomeruli in the *db/db* group showed noticeable swelling, evident glycogen deposition, prominent extracellular matrix deposition by glomerular mesangial cells, and thickening of the basement membrane. Various degrees of improvement were observed in each treatment group. The BA and MIX treatment groups exhibited reduced glomerular volume, a significant reduction in extracellular matrix deposition by glomerular mesangial cells, decreased thickness of the glomerular mesangial basement membrane, and decreased glycogen deposition. However, these changes were not significant, as shown in Figure 3.

### Effect of baicalin and MIX on fibrosis in db/db mice

**FIGURE 4:**
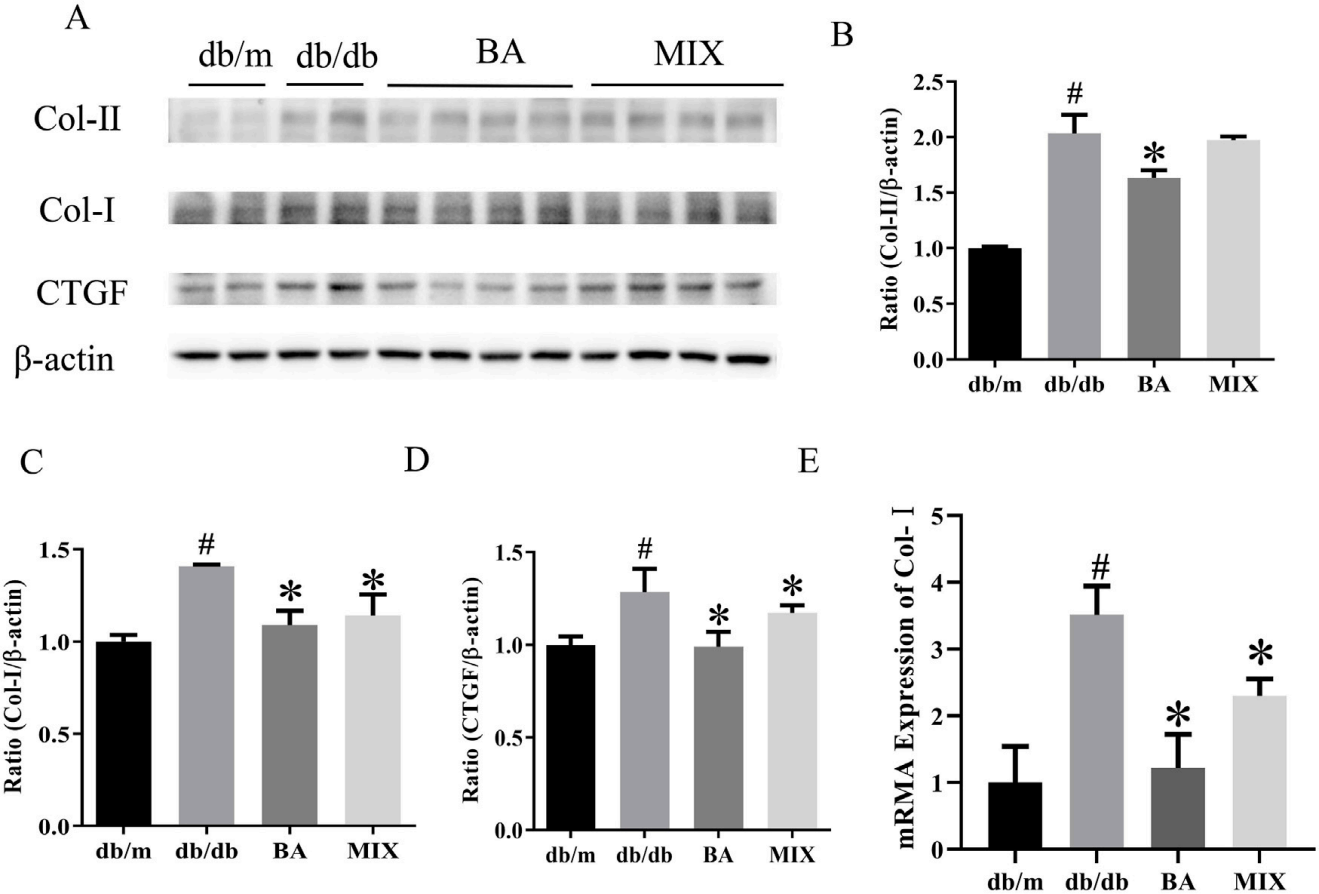
Effects on the expression of fibrotic protein in type 2 diabetic mice **(A)** Immunoblotting bands depicting collagen-II, collagen-I, and CTGF protein levels in mice in each group. **(B)** Expression of collagen-II protein in the mice of each group. **(C)** Expression of collagen-I protein in the mice of each group. **(D)** Expression of the CTGF protein in the mice of each group. **(E)** Relative expression of collagen-I mRNA in each group (n = 6, compared to the db/m group, #*p* < 0.05; compared to the db/db group, *p < 0.05). Abbreviations: HE: hematoxylin and eosin, PAS: periodic acid-Schiff, db/m: normal control group of non-diabetic mice, db/db: model of diabetes, BA: baicalin, MIX: multi-component mixture, Col- (I) collagen I, Col-II: collagen II, CTGF: connective tissue growth factor.

The Western blot results and data analysis are presented in Figure 4. These results demonstrate that, compared to the *db/m* group, the model group exhibited a significant increase in the protein expression levels of CTGF, Col-Ⅰ, and Col-ⅠI. In the treatment groups, both BA and MIX demonstrated varying degrees of improvement in CTGF, Col-Ⅰ, and Col-ⅠI levels. Specifically, the BA treatment group showed significantly reduced expression of Col-I protein compared to that of the *db/db* group. Simultaneously, the MIX treatment group also exhibited a reduction in the expression of Col-I protein, although the difference was not statistically significant. The BA and MIX treatment groups showed significantly decreased expression of Col-I protein compared with that of the model group. Regarding CTGF protein expression, the BA group showed significantly reduced levels compared to those in the *db/db* group, and the MIX treatment group also showed a reduction, although this was not statistically significant. These results indicated that BA and MIX could effectively ameliorate renal fibrosis in diabetic mice, with BA demonstrating a slightly more pronounced effect.

RT-PCR revealed that, compared to the *db/m* group, Col-Ⅰ mRNA expression levels were markedly elevated in the *db/db* group, indicating severe fibrosis in the *db/db* group. In contrast, the BA and MIX treatment groups showed varying degrees of improvement in Col-I gene levels. The BA treatment group exhibited the most pronounced reduction in gene expression. Simultaneously, the MIX treatment group demonstrated a decrease in fibrosis, although not as extensively as that in the BA treatment group.

The findings from the transcriptional to translational levels of fibrotic factors clearly demonstrate a substantial increase in these factors in diabetic organisms. BA emerged as an effective agent for improving diabetes-induced renal fibrosis, thereby slowing the progression of diabetic kidney sclerosis.

### Baicalin and MIX inhibited the TGF-β/Smads signaling pathway in the renal tissues of db/db mice

**FIGURE 5:**
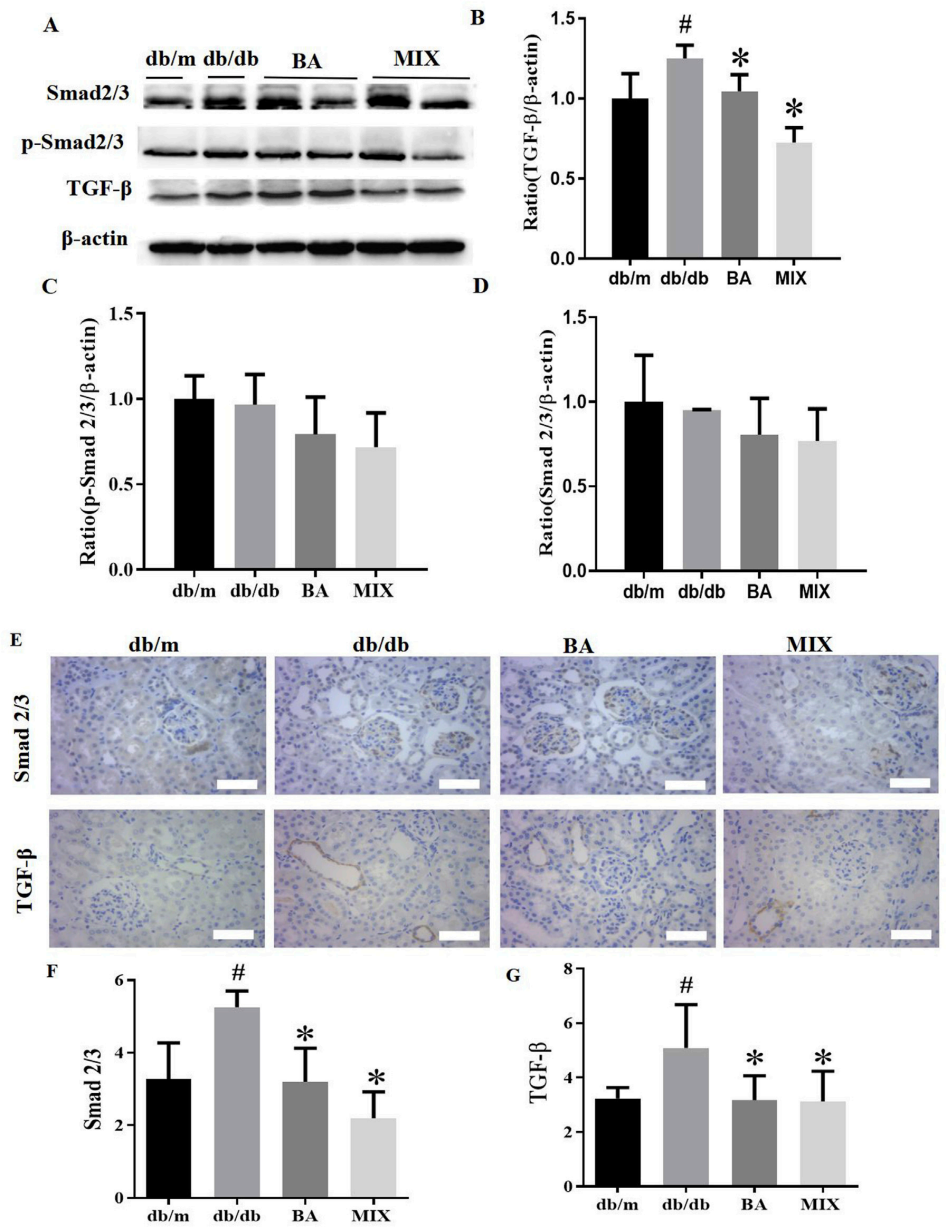
Effects indicating that baicalin and *Scutellaria baicalensis* mixture can inhibit the TGF-β/Smads signaling pathway in the renal tissues of HG mice **(A)** Immunoblotting bands depicting TGF-β, Smad2/3, and p- Smad2/3 protein levels in the mice from each group. **(B)** Expression levels of TGF-β protein in mice from each group. **(C)** Expression levels of p-Smad2/3 protein in mice from each group. **(D)** Expression levels of Smad2/3 protein in the mice from each group. **(E)** Photomicrographs of Smad2/3 and TGF-β staining of the kidney sections from each group, observed under a light microscope (×400). **(F)** Quantitative statistical plot of Smad2/3 in kidney sections. **(G)** Quantitative statistical plot of TGF-β in kidney sections (n = 6, compared to the *db/m* group, #*p* < 0.05; compared to the *db/db* group, *p < 0.05). Abbreviations: HE: hematoxylin and eosin, PAS: periodic acid-Schiff, db/m: normal control group of non-diabetic mice, db/db: model of diabetes, BA: baicalin, MIX: multi-component mixture, Col- (I) collagen, I Col-II: collagen II, TGF-β: transforming growth factor-β.

Our study evaluated the expression of TGF-β, Smad2/3, and p-Smad2/3 by Western blots and immunohistochemistry staining (Figure 5). Western blot results and data analysis are presented in Figures 5A–D. These results demonstrate that, compared to the *db/m* group, the *db/db* group exhibited a significant increase in protein expression levels of TGF-β, Smad2/3, and p-Smad2/3. In the administration groups, BA and MIX demonstrated varying degrees of improvement in TGF-β, Smad2/3 and p-Smad2/3 protein levels. Specifically, the MIX treatment group significantly reduced the expression of TGF-β, Smad2/3, and p-Smad2/3 proteins compared to that of the *db/db* group. Concurrently, the BA treatment group also showed a reduction in the expression of TGF-β, Smad2/3, and p-Smad2/3 proteins, although it was not statistically significant. These results indicate that BA and MIX can inhibit the TGF-β/Smads signaling pathway in the renal tissues of *db/db* mice, with MIX demonstrating a slightly more pronounced effect.

TGF-ß and Smad2/3 were weakly expressed in the kidneys of *db/m* mice but showed strong expression in the kidneys of *db/db* mice, with brown staining detected predominantly in glomerular epithelial cells and renal tubules. The levels of TGF-ß and Smad2/3 were significantly reduced in both treatment groups compared to the *db/db* group, although they remained higher than those in the *db/m* group (Figures 5E–G). Meanwhile, no significant differences were observed between the BA and MIX treatment groups, indicating that both BA and MIX reduced the abundance of TGF-β and Smad2/3 proteins in the renal tissues of *db/db* mice.

## Discussion

DKD is a chronic microvascular disease caused by sustained hyperglycemia. The incidence of DKD in patients with diabetes exceeds 40% and is a key factor in the development of end-stage renal disease (Chen et al., 2022). Without timely treatment, it can lead to kidney failure, significantly increasing patient disability and mortality rates. With the improvement of living standards in recent years, the incidence and mortality of DKD have been on the rise. Currently, the treatment of DKD mainly involves comprehensive therapies, such as glycemic control, lipid regulation, anti-inflammatory drugs, and calcium channel blockers. However, the treatment outcomes are not entirely satisfactory. Therefore, actively exploring new therapeutic drugs for DKD holds significant importance. DKD in traditional Chinese medicine belongs to diabetes syndrome, the main pathogenesis of which is Qi-yin deficiency, stasis, and turbidity-poison stopping. *Scutellaria baicalensis* is known for its high content of active components. These components have been shown to reduce mouse body weight, regulate blood lipid metabolism, and significantly improve insulin sensitivity (Miao et al., 2023).

Renal tissue fibrosis is a pathological, morphological, and structural change that occurs in the early stage of DKD and progressively aggravates, which is an important reason for the continuous deterioration of renal function. In studies of the pathogenesis of DKD, researchers have found alterations in cytokine levels in such patients, which can exacerbate renal function loss. Examples include the activation of signaling pathways such as TGF-β, Smad, and p38MAPK.

To investigate the effects of BA and MIX (WGN, BA, and WGS), we used a spontaneous mouse model of type 2 diabetes (db/db) to assess how these interventions impact diabetic kidney disease (DKD) in mice. These components improve glomerular filtration function, reduce the kidney enlargement index, and improve kidney function. They alleviate interstitial fibrosis, reduce glomerular volume, and inhibit the formation of the glomerular mesangial matrix (Hu et al., 2024). Reduces the synthesis of Col-I at both the gene and protein levels. Decreases the production of CTGF, an early marker of fibrosis. TGF-β1 is a universal cytokine in mammalian cells; it can induce the transcription expression and deposition of extracellular matrix genes and accelerate tissue fibrosis. Col-I and Col-II are essential components of the extracellular matrix (Hu et al., 2018; Chen et al., 2021). Multiple signaling pathways regulate the transformation of renal tubular epithelial cells into fibroblasts, with the TGF-β1/Smads pathway playing an important role (Gewin, 2020). The expression of Smads is regulated by TGF-β1, with Smad2 and Smad3 being most closely related to renal fibrosis. TGF-β1 can induce their polymerization (Smad2/3), which shifts to the nucleus and induces excessive expression of the extracellular matrix to promote tissue fibrosis (Budi et al., 2021; Walton et al., 2017).

This study showed that MIX enhanced lipid profiles and renal function by improving renal tubular dilation, restoring the tight arrangement of renal structures, and reducing glomerulosclerosis, basal membrane thickening, and glycogen deposition. These effects were achieved by reducing the protein and gene expressions of Col-II, Col-I, and CTGF. This indicates that inhibiting the TGF-β1/Smad2/3 pathway may be an important molecular mechanism by which MIX inhibits renal fibrosis in *db/db* mice. The results suggest that BA and MIX play protective roles against DKD. The therapeutic effect of MIX was superior to that of BA. MIX significantly reduced body weight, improved insulin sensitivity, lowered the renal hypertrophy index, and reduced BUN levels and the urine protein/creatinine ratio. MIX inhibited the TGF-β/Smads signaling pathway, thus alleviating renal fibrosis.

In the study, we focused on investigating the effects of BA and MIX, specifically examining the potential of MIX in alleviating fibrosis associated with DKD. BA is one of the primary active flavonoid compounds in S. *baicalensis* and has demonstrated beneficial effects in reducing fibrosis, inflammation, and oxidative stress in DKD, making it a promising candidate for further development and application. WGN and WGS were a kind of flavonoids, which have many pharmacological effects, such as anti-oxidation, antiinflammatory and anti-fibrosis. WGN treats DKD by in mitigating tubulointerstitial fibrosis and renal tubular cell injury via regulating PI3K/Akt/NF-κB signaling pathway-mediated autophagy and inflammation (Lei et al., 2021), glomerulopathy and podocyte injury by regulating Bcl-2-mediated crosstalk between autophagy and apoptosis (Liu et al., 2022). WGS can inhibit inflammation, oxidative stress regulates renal endothelial injury (Wang YR. et al., 2022). Although the results of the study suggest that WGN and WGS have a certain application in the treatment of DKD. However, the main purpose of this study was to evaluate the effect of MIX combination therapy. And focused on investigating the effects of BA and MIX, specifically examining the potential of MIX in alleviating fibrosis associated with DKD with the aim of advancing the development of MIX.

The value of comparing the individual effects of WGN and WGS with MIX is also recognized (Liu et al., 2022; Wang YR. et al., 2022; Lei et al., 2021), as this would provide insights into potential synergistic effects. Future studies will explore the interactions among BA, WGN, and WGS in more detail. To this end, additional experiments will be conducted to compare WGN and WGS with MIX, in order to better understand each component’s contribution and the overall effectiveness of MIX. It remains unclear how MIX regulates downstream genes and proteins in the TGF-β1/Smad2/3 pathway. Additionally, the impact of MIX on fibrosis in DKD mice involves multiple pathways, requiring further investigation.

## Data Availability

The original contributions presented in the study are included in the article/supplementary material, further inquiries can be directed to the corresponding authors.
